# Exploring the use of observational tools for advancing patient safety learning among preregistration healthcare students: a scoping review using the 3P model of teaching and learning

**DOI:** 10.1186/s12909-026-09275-8

**Published:** 2026-04-23

**Authors:** R. Upreti Oli, T. R. L. Griffiths, R. I. Norman, E. S. Anderson

**Affiliations:** Leicester Medical School, University of Leicester, Leicester, England

**Keywords:** Healthcare students, Observational learning, Patient safety, Safety tool, Theory, 3P model

## Abstract

**Background:**

Patient safety is a global health priority and a fundamental part of healthcare curricula worldwide. While teaching medical students on theoretical components is straightforward, students may fail to prioritise patient safety due to an incomplete understanding and experience of the pressures of front-line care. Using an observational tool for patient safety learning in real clinical environments appears to help students engage with the significance of safe practice. This review seeks to identify learning approaches that use observational safety tools in practice.

**Methods:**

This scoping review follows the JBI Evidence Synthesis Template. We searched Medline, CINAHL, Scopus, Web of Science, and The Cochrane Library for relevant papers published from 2009 to June 2024. Papers were appraised for quality indicators and pedagogical theory. They were synthesised using Bigg’s 3P theoretical model into presage (teaching setup), process (teaching), and product (evaluation and assessment) factors.

**Results:**

Of an initial 10,203 articles, only eight met the search criteria. The selected studies were conducted in North America (five) or Europe (three). The studies involved medical and nursing students in mid-to-late training and were for uni or interprofessional learning. Most studies involved a few representative students (range 11–368). They were conducted in acute clinical settings, including wards and theatres. All studies claimed the use of a safety tool to advance students’ learning, and many involved monitoring real-time clinical practice. The authors reported that the students gained new knowledge, identified errors, valued learning in a team and felt better prepared to speak up for errors. The studies failed to fully describe presage factors for setup challenges and issues. They lacked rigour and adequate theoretical explanations about how learning took place (process). As the work was mainly descriptive and atheoretical, assessment outputs were lacking, but evaluations were described.

**Conclusions:**

Observational patient safety learning tools used in practice benefit students’ understanding of patient safety, advancing their preparation for practice and remain under-studied and under-developed. The studies were often in their pilot development stages and hence involved few students. Further research on the use of patient safety education tools, used in practice, is urgently required to help educators with teaching design, delivery and assessment.

**Supplementary Information:**

The online version contains supplementary material available at 10.1186/s12909-026-09275-8.

## Introduction

Patient safety continues to be an important goal for all healthcare systems [1, 2]. While tackling the myriad of potential patient safety factors remains hard [3], pre-registration healthcare students must understand the main concepts and today’s challenges for safe practice. The World Health Organisation (WHO) has led the way in producing educational interventions for all pre-to-post-qualified healthcare practitioners to prepare them to deliver safe care [4, 5]. In the UK, the General Medical Council (GMC) offer a patient safety framework [6]. This is mirrored globally, for example, in the USA [7, 8], Canada [9], and Australia [10]. Patient safety learning is expected for all professionals throughout their careers, and today, the emphasis is on understanding healthcare systems, human factors and the importance of a collaborative and supportive culture [11–14].

We still do not know the most effective ways of teaching pre-registration students about the importance of safety in preparation for practice, and there are wide variations in teaching approaches [15–17]. While it is possible to impart knowledge about human factors and quality improvement, students are naive about human vulnerability in today’s fast-paced frontline clinical settings [18–21]. Educators can struggle to make the important link between the significant theory and safe practice readiness. While students may not prioritise patient safety learning, they are aware of what happens in practice, as they see care delivery through their lens as outsiders to the system, informed by best-practice prior learning [22]. However, newly qualified practitioners and senior students can quickly spot poor standards of care [23, 24], identify and report poor and positive practices and yet their voice is rarely considered [25, 26]. Using supportive tools for this learning has been reported as one way to advance students’ competence, mirroring aviation approaches to observing real-time practice shown to advance safety [27]. Observational learning is known to be very powerful [28], with theorists showing how learners observe the behaviour of a role model, and transform the information into a symbolic concept, which is transformed into motivated practice [29].

In this review, we set out to further explore how undergraduate/pre-registration healthcare students’ learning on patient safety has been advanced by being observers in practice supported by tools or resources that help focus students’ appreciation of the safety issues. We use a scoping review methodology because using tools to advance learning is new and developing. We reflected on ways to assess papers for teaching design and found the Biggs’ 3P model most helpful because it considers the teacher and students factors need to set up learning events (Presage Factors), what happens in the learning event (Process Factors) and the impact of the learning event on students to guide educators in the replication of these teaching approaches [30].

## Methods

The scoping review followed the JBI Preferred Reporting Items for Systematic Reviews and Meta-Analyses extension for Scoping Reviews Checklist [31]. The protocol was registered in the Figshare database of the University of Leicester [32].

The scoping review asked, “How have observational tools advanced healthcare student learning on patient safety?” The sub-questions were:

- How are the tools designed, used and evaluated?
- How does this method advance patient safety learning?
- What are the problems identified while using a tool?
- Has theory been applied to explain observational patient safety learning?

### Defining the context

The population, concept and context of the study were identified and guided by a librarian research consultant (Table 1). The study population included healthcare undergraduate/pre-registration students and new graduates working/learning in acute hospital settings. The concepts were observational tools or resources that were used in real-time in practice for patient safety learning. A tool was perceived as any resource or learning framework used to support patient safety learning. The focus of the learning was on patient safety relating to human factors, systems and environment, not clinical decision-making.

### Search strategy

We looked for primary, peer-reviewed pedagogical international research papers in English from 2009 to June 2024. The start date aligns with the WHO’s first publication of a patient safety curriculum [4]. Exclusion criteria included grey literature, non-peer-reviewed papers and social care students. The search was conducted in Medline, CINAHL, Scopus, Web of Science and The Cochrane Library (Table 1).

**Table 1:** List of search terms

Population		Concept		Context
“undergraduate” OR “pre-registration” OR “foundation doctor” OR “internship” OR “preceptor nurse” OR “nursing student” OR “pharmacy student” OR “radiography student” OR “midwifery students” OR “operating department practitioners’ students” OR “healthcare students” OR “medical students”	AND	“instruments” OR “tools” OR “checklist” OR “applications” OR “apps” OR “policy” OR “framework” OR “intervention” OR “software”	AND	“Patient safety” OR “medical error” OR “quality improvement” OR “errors reporting” OR “learning from errors” OR “human factors engineering” OR “root cause analysis” OR “system thinking” OR “incident reporting” OR “near miss” OR “error disclosure” OR “safety culture”.

### Study selection

**Fig. 1:**
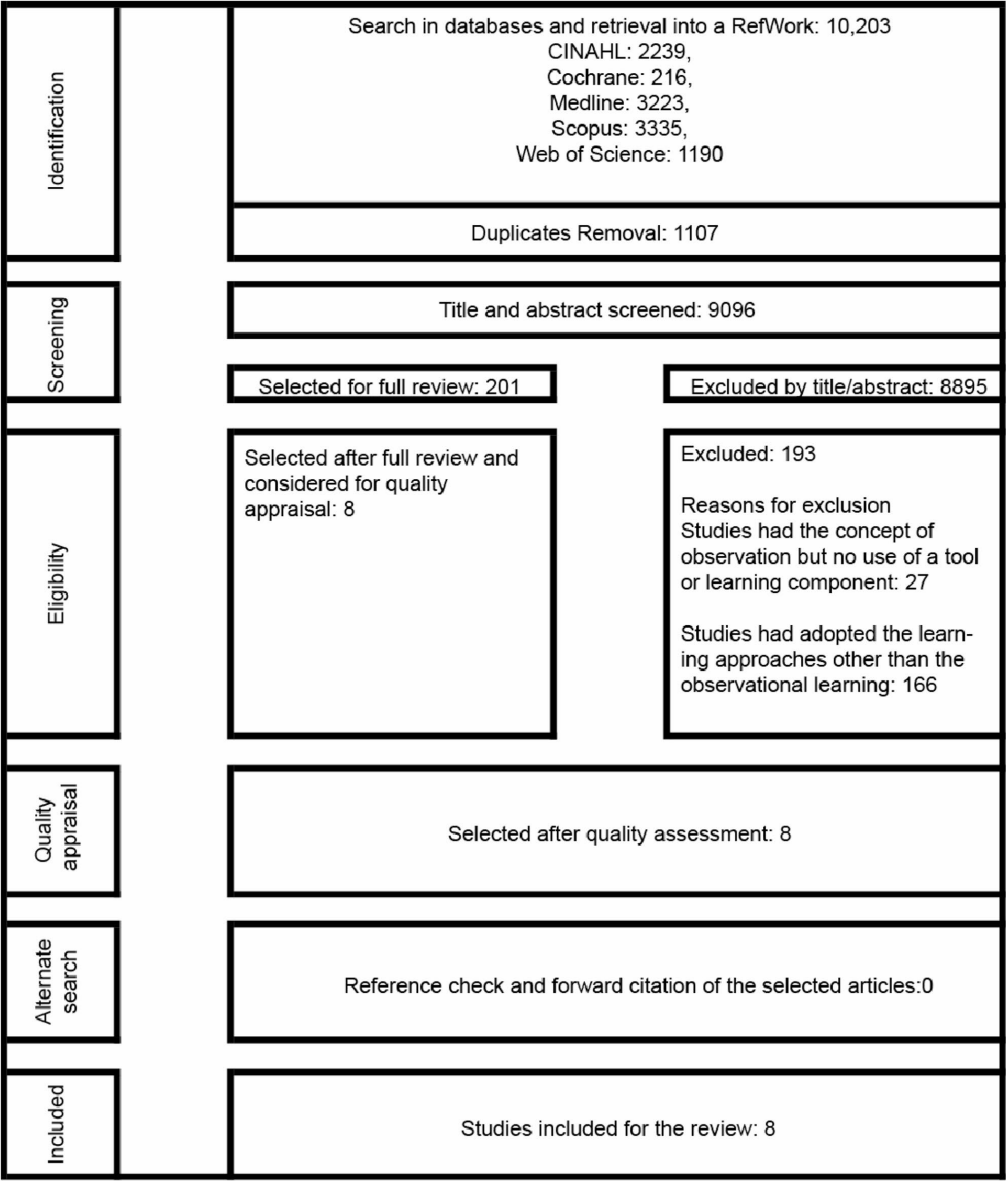
Flowchart of selection process

The search identified 10,203 papers leaving 9096 for screening (Fig. 1). The preliminary screening for the title and abstract was carried out by (RUO) and was cross-checked (ESA and TRLG). We tested the search using one known paper [27]. The search output was managed using RefWorks software.

### Review of eligible articles

The first analysis identified 201 studies for full-text analysis against all aspects of the eligibility criteria. This left 35 papers for further debate and discussion (RU, LG, RIN, ESA) and of them, eight met the inclusion criteria. While we included Quality Improvement (QI) in the initial search, we excluded QI plan-do-study-act studies that did not include a practice-aligned observation learning tool. Several papers that used the SBAR (Situation, Background, Assessment, Recommendation) communication tool were excluded as they were used in classroom settings. Any disagreement that arose between the reviewers at each stage of the selection process was resolved through discussion. The final set of eight included papers (IN papers) was entered into an MS Excel data extraction template and charted (Table 2). References and citations of the final IN papers were screened for missing articles. Likewise, the methodological rigour was assessed by using the Best Evidence Medical Education (BEME) checklist [33], and a theoretical quality appraisal tool was used to assess how theory guided the study design [34].

### Collating, summarising and reporting the results

The synthesis of the review was conceptually guided by Biggs’ systems approach to teaching and learning [30]. The 3P model provides a framework to understand the pre-delivery teaching set-up (presage), the learning delivery (process) and the outcomes (product) of the learning. The IN papers were aligned with this theory and analysed using reflexive thematic analysis [35].

## Results

### Description of selected papers

Eight studies were published from 2011 to 2022, five from North America and three from Europe [27, 36–42]. One study analysed the use of a general safety learning tool in five European countries [41] (Table 2). All of the IN papers scored above the minimum cut-off of seven when screened for research quality using the BEME checklist [33] (Appendix I).

The students’ years of study ranged from the early first year to the final year, from medical to nursing schools (Table 2). Two studies were conducted as a part of the core curriculum in line with accreditation norms [36, 38]. Two were conducted during the students’ electives [27, 37]; one was an audit [39]; one utilised students’ outpatient rotation for the hospitals’ quality improvement project [42]. Only one study reported the gender of participating students – 64% were females [37]; none mentioned their age. One study reported the timing of observation – 7 a.m. to 4 p.m. during the weekdays [39].

**Table 2:** Description of selected articles

SN/ citation no.	Citation	Observation Safety Tool for Learning	Participants and Numbers	Duration/No of observation	Place of study	Scope of Study
1/ [27]	Anderson ES, Griffiths TRL, Forey T, Wobi F, Norman RI, Martin G. Developing Healthcare Team Observations for Patient Safety (HTOPS): senior medical students capture everyday clinical moments. Pilot Feasibility Stud. 2021;7(1):164.	Healthcare Team Observation of Patient Safety (HTOPS)	34 Final-year medical students in 3 cohorts	6 days of observation within a 3-week module in one year; continued up to 3 years 917 cases of observation in total	Leicester Medical School, UK	Patient safety education
2/ [36]	Beekman M, Emani VK, Wolford R, Hanson K, Wickham G, Aiyer M. Patient Safety Morning Report: Innovation in Teaching Core Patient Safety Principles to Third-Year Medical Students. J Med Educ Curric Dev. 2019;6:2382120519842539.	Safety concern, Action taken, Failure causes of the event, and Effects (SAFE) Framework	75 Third-year medical students	Each student presented one observation within 8 weeks of posting 75 cases in total	University of Illinois College of Medicine Peoria, USA	Patient safety education
3/ [37]	Bennett SA. Aviation’s Normal Operations Safety Audit: a safety management and educational tool for health care? Results of a small-scale trial. Risk Manag Healthc Policy. 2017;10: 147–165	Threat and Error Assessment and Management Worksheet (TEAM – W)	11 Fifth-year medical students in two groups	6 days of observation within a 2 week module/ 359 threats and 86 errors identified	School of Business, University of Leicester, UK	Safety management and educational benefits
4/ [38]	Dudas RA, Bundy DG, Miller MR, Barone M. Can teaching medical students to investigate medication errors change their attitudes towards patient safety? BMJ Qual Saf. 2011;20(4):319 − 25	Learning from Defect (LFD)	108 s, third, and fourth-year medical students	25 student groups identified one issue per group/ total issues: 25	Johns Hopkins University School of Medicine, Baltimore, USA	Medication error
5/ [39]	Logan CA, Cressey BD, Wu RY, Janicki AJ, Chen CX, Bolourchi ML et al. Monitoring universal protocol compliance through real-time clandestine observation by medical students results in performance improvement. J Surg Educ. 2012;69(1):41 − 6.	Compliance Checklist based on the Tufts Medical Center’s Presurgical Checklist	14 Medical students in two batches	Year 1: 98 surgeries/8 students; Year 2: 100 surgeries/ 6 students (one week of observation)	Tufts University Medical Center, Boston, USA	Universal protocol compliance
6/ [40]	Spence J, Goodwin B, Enns C, Vecherya N, Dean H. 2012. Patient safety education: an exploration of student-driven contextual learning. J Nurs Educ. 51(8):466 − 70	WHO Surgical Safety Checklist (45)	130 First-year and second-year medical and third-year nursing students	One IPE pair recorded one WHO checklist/ 65 incidents in total	University of Manitoba, Winnipeg, Canada	Surgical safety practices
7/ [41]	Steven A, Pearson P, Turunen H, Myhre K, Sasso L, Vizcaya-Moreno MF et al. Development of an International Tool for Students to Record and Reflect on Patient Safety Learning Experiences. Nurse Educ. 2022;47(3):E62-e67.	SLIPPS (Sharing Learning from Practice of Patient Safety) Learning Event Recording Tool (SLERT)	368 Nursing students	368 student reports on safety concerns	Five European Countries: UK, Finland, Italy, Spain and Norway	Patient safety education
8/ [42]	Thompson D, Bowdey L, Brett M, Cheek J. Using medical student observers of infection prevention, hand hygiene, and injection safety in outpatient settings: A cross-sectional survey. Am J Infect Control. 2016;44(4):374 − 80.	Tool guided by the Centers for Disease Control and Prevention (43) on Infection Prevention and other hand hygiene audit tools	Second-year medical students (number not specified)	Minimum 10 injections per site and an average of 22 hand hygiene opportunities per site/ 163 injection safety and 330 hand hygiene in total	University of New Mexico School of Medicine, Mexico	Infection prevention, hand hygiene, injection

### Description of the tools used

All papers explained their objectives explicitly and aimed to promote student learning to advance understanding of the broader concepts of patient safety (Table 2). Two studies focused on surgical safety [39, 40], two on adapting aviation’s safety management tools [27, 37], one on medication error [38], one on infection prevention [42], one on paediatric safety domains [36] and one on unspecified hospital contexts [41].

Two studies designed the teaching tool using standards from aviation [27, 37]. One study [37] directly used the terminology of aviation audits, Normal Operations Safety Audit, using a template to record observed safety ‘Threats’ and/or ‘Errors’ in healthcare delivery. These concepts were further advanced using the aviation tool for healthcare and led to the design of the Healthcare Team Observations for Patient Safety tool (HTOPS) using participatory action research (PAR) and asked students to record safety concerns for human factors, systems, and environment in an app [27].

Beekman et al. [36] developed a SAFE framework (Safety concern, Action taken, Failure, Effects) based on Kolb’s Experiential Learning model [43]. Dundas et al. [38] designed a ‘Learning from Defect (LFD)’ tool similar to a root cause analysis to identify and analyse medication errors in paediatrics. The tool had three sections: a description of the event, an explanation of the reason based on broader safety elements and lastly the proposed solution to the problem to capture both positive and negative factors. In the Spence et al. study [40], the WHO surgical safety checklist tool [44] was used by nursing and medical students who documented the observations of different surgeries. Also in surgery, Logan et al. [39] used a Universal Protocol Compliance for student observations of surgical care. The researchers prepared a compliance checklist based on the Tufts Medical Centre’s pre-surgical checklist, which consisted of surgical safety practice teams practising pre-surgical incision, for time out, attentiveness, recording, teamwork, and communication.

A multinational three-year European project developed, translated, and pretested the SLIPPS (Sharing Learning from Practice of Patient Safety) Learning Event Recording Tool (SLERT) based on the international literature, theory, and multidisciplinary experience [41]. The tool consisted of instructions, sections allowing nursing students to describe and reflect on safety concerns and a final section related to student demographics and characteristics of the event observed. Thompson et al. [42] designed a new tool guided by the elements of the Centers for Disease Control and Prevention (CDC), to record safety practices related to infection prevention, hand hygiene, and injection safety [45].

### Presage factors

We explored how the teaching was set up for learning (Table 3). Four studies were conducted in clinical units, including operating rooms/theatres, wards, clinics, and mental health facilities [27, 37, 39, 40]. Two studies were within paediatric inpatient and outpatient settings [36, 38], and one study was based in 15 different outpatient settings [42]. The international study aimed to design a tool for wide application in nursing practice, failing to specify the clinical context(s) where the tool was tested, but they were in the hospital [41].

**Table 3:** Presage factors of observational learning using the safety tools

SN/ Citation No.	Alignment in Curriculum	Clinical Setting of Observation	Clinical Preparation	Teacher Involvement and Preparation	Student preparation for the learning	Resources
1/ [27]	Aligned to the annual spring special study module	Wards, theatres, and clinics	Clinical areas selected and briefed on receiving students by academic clinical leads	Involved local hospital patient safety leads and clinical teachers familiar with patient safety; they connected students to the clinical practitioners	The students who used the tool were in their final years and were asked to build upon earlier theoretical and simulated safety learning. They were prepared for data collection, reflection of learning, observation, and use of tools	Initially used the paper tool; in the third-year observation app was designed
2/ [36]	Conducted as a part of core clerkship taken by all students	Inpatient and outpatient paediatric units	-	Interdisciplinary faculties with patient safety expertise reviewed safety cases identified by students, re-categorised them into the safety domains, provided feedback to the students and reported the safety events to the concerned unit	Students were oriented to the tool, and asked to complete the online introductory and advanced course on patient safety and quality improvement from the Institute of Healthcare Improvement. The students attended in-person training on team communication, human factors, and root-cause analysis of errors	-
3/ [37]	During the student-selected components module	A mental health facility, a fracture clinic (acute), a urology ward, and general and vascular surgery theatres	Observers verbally explained the purpose of their study to the observes (clinical practitioners) as the NHS waived the written consent	Convenors signed off the students’ competence log-book and safety tools recordings, attended students’ debriefings, took notes on students’ observations	One day training on the tool, video on recognising risk and patient safety, discussion session with students	Clipboards, student IDs
4/ [38]	Introduced as an experiential patient safety curriculum as a part of core clinical clerkship	Paediatric - ambulatory and inpatient setting	-	Faculties explained the tool to the students, presented an example case and reviewed the cases submitted by students	Students attended a nine-week mandatory curriculum run over one year consisting of an introduction video followed by two one-hour large group (23–25 students) sessions and a small group (*n* = 4–5) self-directed observation exercise.	-
5/ [39]	-	Operating room	The operating room coordinator placed the students across different surgeries for the secret observation	Chief clinicians were involved in designing the compliance standards, validating and sharing the results with other key clinicians in the hospital and counselling the surgeons in complying with the surgical guidelines	-	-
6/ [40]	Aligned to the students’ surgical rotation	Operating room	Operating room nurse educators and managers matched the students’ availability with the operating room capacity	Surgery course instructors and peri-operative nurse educators provided orientation to nursing and medical students, respectively, matched the IPE pairs and assigned them for the observation	Students were introduced to the operating room procedure, the WHO checklist, patient safety, and adverse events following the WHO safety curriculum.	-
7/ [41]	Each participating country implemented this tool as an adjunct to its existing pedagogical resources	Tool designed to be used across wider nursing settings, from hospital to community; not mentioned where it was tested	-	-	-	An electronic web-based or hand-written tool for the observation
8/ [42]	As a part of students’ outpatient rotation	Outpatient clinical practice units	-	Preceptors provided students with permissions and managing the breach in safety protocol identified by students	Students were oriented to learning over a 6-week teaching plan delivered online and face-to-face, on infection prevention resources, audit, observation, tools, lectures, and discussion.	-

One study motivated the students to take part by awarding 5% points to their final grade [38]. Several studies outlined how to prepare students for learning in a clinical environment. The studies discussed extensive training on patient safety and the tool for data collection in preparing the students for their work using tools [27, 36–38, 40, 42] (Table 3), however, in all studies, there were no pre-measurements of the student’s prior patient-safety learning. There was no outline on what resources teachers needed to set up these studies, with some using electronic apps or web-based tools while others provided paper-based tools along with ID cards and clipboards. No study mentioned resource requirements costs.

Very few of the studies reported the teachers’ involvement and expertise. Anderson et al. [27] involved wider stakeholders including aviation experts, healthcare professionals, academics and local safety leads in designing and refining the tool. In some studies, the clinical teachers were involved in briefing and preparing the clinical team for student-led observation. In these studies, the clinical faculty facilitated the student placement [37, 39] or provided permission for the study but had limited involvement [40] (Table 3).

### Process factors

Five papers articulated theory to guide their project [27, 36–38, 41] relating to experiential learning, action learning [27, 37], actor-network theory and system theory [37], reflective practice and situated learning [41] (Table 4). The theories adopted were often only briefly mentioned and failed to explore how a particular approach had enhanced student learning.

All the authors described the students’ observation process explicitly. The students had active and immersive observations related to patient safety issues in the acute clinical context, which they recorded for reflection on the tool provided. Some students observed both positive and negative safety issues [27, 38, 41] and all presented the observed incidents to either the clinical teams or their teachers (Table 4). The role of the teacher in all eight studies was clearly described. In one study, clinical teachers did a categorisation of the safety domains of the issues identified by the students, the difference was later statistically analysed in the quantitative evaluation [36]. They reported serious concerns identified by students to the clinical teams [42]; and did the validation [36, 39] of the observation.

**Table 4:** Process factors of observational learning using the safety tools

SN/ Citation No.	Theoretical Integration of Learning Process	Student Observation Learning Process
1/ [27]	Participatory action research, experiential learning	Students reported both anonymised positive and negative safe practices on paper or electronic tools, and the outcomes were fed back to the healthcare teams.
2/ [36]	Kolb’s Model for Experiential Learning	Each group of 10–12 students attended two patient safety morning report sessions where every student presented an incident with a concern about safety during their eight-week-long paediatric rotation. They used six safety domains (human factors, systems, teamworking, communication, clinical risk management, and engaging patients) to explore invasive procedures, infection, and medication safety with an additional unspecified ‘other’, and clinical teachers validated student observations.
3/ [37]	Immersive/experiential learning and action learning, actor-network theory, systems theory	Medical students in small groups (*n* = 2–3) rotated across a range of clinical settings, and observed practice using a paper Assessment and Management Worksheet (TEAM–W).
4/ [38]	Experiential learning grounded in the adult learning principle	Each observing student group used the tool to identify, analyse and organise the errors observed during their clinical postings and present the findings to the larger group in a classroom-based session.
5/ [39]	-	The study reported 6–8 student observations from a hundred day-time surgeries over one week. Students completed the checklist and provided recommendations for improvement. Practitioners compared the student observation with that of chart staff audit reviews.
6/ [40]	-	The observations were completed by an interprofessional student pair observing together in the operating room during sign-in, time-out, and sign-out. Each student pair submitted one checklist along with the observations of the lapses they noted which were labelled by the investigators as errors or adverse events.
7/ [41]	Theory of reflective practice, experiential and situated learning,	Students observed positive or negative safety events of their choice from a long list of safety concerns either on a handwritten or electronic mobile/laptop-based tool.
8/ [42]	-	Students interviewed medical staff using a 92–item checklist on infection control from administrative policies to training, occupational health, reporting, clinical cleaning guidance, and processing of reusable devices. In addition, students anonymously observed injection safety and hand hygiene using tailored tools which were then compared with that of self–reported behaviour during the medical staff interviews. Any serious concerns were reported to the clinical preceptors by the student supervisors.

### Product Factors

The research or evaluation study designs used a wide range of theoretical approaches (Table 5). These were mainly qualitative, involving student interviews and focus groups, often using clinicians to measure the impact along with the student reflection notes recorded in the tools [27, 37, 40, 41]. Two studies evaluated learning using a cross-sectional survey with scored questions on a Likert Scale, with additional free text student comments [36, 38]. Most studies contained post-observation student debriefing and reflection, where students presented their results [27, 36–39] or submitted reflective reports [39, 40, 42] to the clinical teams or the patient safety educational leads. Two studies did not mention whether they researched or evaluated the students’ learning experience, but concluded that the student findings could be utilised to improve practice [39, 42] (Table 5).

**Table 5:** Product factors of observational learning using the safety tools

SN/ Citation No.	Learning Assessment	Impact evaluation	Impact of observational tools on patient safety learning	Limitation
1/ [27]	Formative (Students feedback on the learning in a presentation)	Interviews of students and clinical staff	All students had advanced their patient safety understanding; “*Makes you more vigilant while observing others making mistakes*”, pg 15; Students observed incorrect surgical whiteboard and consultant-nurse communication; Observations fed into plans for improving students’ practice; Students tagged it as a good method as it helped them (students) see the totality of the practice.	Hawthorne effect, students’ limited skill in research communication, the threat felt by first-line staff
2/ [36]	Formative (Two students led short didactic sessions on a key safety concept followed by a discussion of the patient safety cases)	An 11-item questionnaire with a 1–5 Likert-type scale along with adding any free text feedback at the end	100% of students reported a favourable gain in the knowledge base; 94% and 98% of students reported they were able to recognize components of error and safety lapses respectively; 98% appreciated the value of communication; 99% felt safe, 94% prepared to discuss safety breaches; “*Appreciate the non-judgmental environment to discuss and prepared for future safety incidents*” pg 6; 89% said they could overcome safety issues in the future; 83% wanted more of this learning in the future, 71% asked to do this in other clinical settings, and 95% felt worthwhile.	Students did not identify system and family as safety domains, study did not assess the prior learning of students
3/ [37]	Formative (Case study-based interim debriefing and a final PowerPoint presentation)	Interviews among students	All students claimed study improved their awareness of patient safety; Improved situational awareness and observation; identified threats and errors; Improved communication; Better self-confidence; Students recommended using the electronic version of the tool; the majority found this method useful.	Hawthorne effect, observer bias, experimenter bias, cognitive overload, lack of accuracy among student observers
4/ [38]	Formative (Small group presentation in the class followed by discussion)	A brief survey instrument using a subset of items from the Safety Attitudes Questionnaire along with additional questions developed by the investigator team using a retrospective pre-post design	Students were able to identify the positive and negative contributory factors of errors observed and suggested preventive measures; Could identify others making the error; Increased appreciation of teamwork and communication; Felt comfortable reporting the problem to the concerned authority; Agreed they might make errors in future, they won’t have difficulty discussing with seniors, Recommended to continue in clerkship.	Recall bias, effort justification bias, no measurement of prior learning, and not sure about the durability of attitudinal changes
5/ [39]	-	Not mentioned how the student learning was evaluated	Students observed errors or non-compliance with the checklist; Noticed lack of team communication during the surgery, e.g., not displaying the image in certain surgical cases, no team introduction.	Restricted time of day of observations, drawbacks of direct observation: interrater assessment variability and the cost of hiring staff to carry out the observations
6/ [40]	-	Focus group regarding the observation on the surgical safety practices in the OR and learning on patient safety	“*Being able to see first-hand how and why* *we do these things in healthcare really made me appreciate their* *importance*” pg 469; “*My role changed from a passive observer to an active participant…”* pg 469; *“It was nice to be paired with a medical student…”* pg 468; *“My IPE pair had a way more OR experience than me….”* pg 468; *“….I have something to discuss with my preceptor and OR team*” pg 469.	The research component of the project was voluntary; those who were not selected to complete an OR visitation might have their attitudes toward patient safety influenced
7/ [41]	Formative (Students were asked to brief how they identified the safety cases and write a reflection note of the safety events)	Semi-structured interviews with some selected students.	“Made me reflect on the event in 3-dimensional - think about how I felt, analyse the contributory factor, and remember what I experienced” pg 65; "I chose this event because the nurse displayed a behaviour I wasn’t used to seeing" pg 64; "I want to record every event if there is a recording system,” pg 65; Students suggested that discussing the event was fruitful, and were also frustrated that they had no such platform before this study.	A small number of students, not sure about the longitudinal impact, limited knowledge of students to comment on the observed practice
8/ [42]	-	Not mentioned how the student learning was evaluated	Students observed non-compliant events related to infection prevention;Students recommended quality improvement initiatives based on their observations.	A limited number of outpatient sites included and a limited number of observations at each site, practices and staff might not be representative of a broader range, findings may not be as generalizable, Hawthorne effects among clinical teams being observed

We identified three broader themes related to the outcome of the studies: pedagogical value, wider clinical impact, and acceptability of tools and processes.

#### Pedagogical value

The authors claimed that their studies enhanced student competence in patient safety by supporting students’ identification of errors, valuing the importance of teamwork and communication, their ability to speak up for error, self-reflection on patient safety learning and preparing them for delivering safe care in the future. The details of the extracts and quotes to support these themes are presented in Table 5.

#### Wider clinical impact

The observational tools were found to be capable of highlighting the totality of safety practices, capturing both strengths and weaknesses and promoting the importance of a just culture [27]. Even though many safety practices were in line with the safety standards, students observed weaknesses in the delivery system and recommended QI initiatives based on the observation [42]. One study stated that the direct observations made by students had the potential to reflect the disparity between expected and actual practice [39].

#### Acceptability of this approach

Students recommended this mode of learning [37, 38, 41]. In the quantitative evaluation [36], 83% of the students wanted more of this in the future, 71% asked to repeat the learning in another clinical setting, and 95% felt the learning was worthwhile. The majority of practitioners [27] and students [27, 37] felt comfortable with the observational approaches. The anonymity, confidentiality and acceptance by frontline staff were essential and highly appreciated [27, 41]. Intuitive use was also important, *‘straightforward to use as the navigation was self-explanatory’* [41].

### Challenges

The major issues reported were student observer bias, recall bias, cognitive overload, limited experience of the students regarding clinical knowledge, research communication, and hesitation to observe clinical teams. Students mainly focused on clinical care and missed identifying system factors and other issues such as family engagement, and these omissions were picked up by practitioners [36]. Within the learning context, the student observers from the early training years with limited clinical exposure were helped by working with senior students [40]. Most studies were new initiatives, and there were concerns for inflated outcome reporting attributed to the Hawthorne effect [27, 37], with concerns that the observed clinical staff might be practising to a higher level of competence than normal. The studies were conducted as a snapshot of the event – neither the prior learning nor the future impact was assessed. Studies also reported the restricted timing of the observations, often with voluntary involvement of the students, limited student numbers, lack of digital tools and scheduling issues (Table 5).

## Discussion

This review sets out to identify studies that tested observational safety learning tools for advancing healthcare students’ understanding of patient safety in preparation for practice. All studies were conducted in Western healthcare systems where safety educational priorities are expected. No studies addressed patient safety in developing countries where the challenges of observational training might be different [46]. Most studies were uniprofessional and for medical students only. While team-based collaborations for patient safety were expected, there were missed opportunities for interprofessional learning [5, 22, 47]. The 3P model synthesis helped to highlight what is known about how to design, deliver and manage this teaching.

Concerning Presage (set up) factors, the studies had not considered students’ prior learning, or teacher factors, such as knowledge and the clinical role. Being mainly pilots, studies had yet to be fully embedded within the curriculum. The content was designed to advance safe practice and the focus of the tools varied from medication errors, infection prevention, and safer surgery, to broaden patient safety concepts. None of the studies linked the learning to Safety I, exploring error after mistakes have been made, or Safety II, looking for good practice and avoidance of mistakes [48]. All studies made justifications for the purpose and content of the tool, from adopting existing tools, for example, the WHO surgical checklist [44], to the design of new tools. The tools could be used in a range of inpatient and outpatient clinical and community units; the majority of the studies were tested in acute clinical settings, mainly operating theatres. This could be due to the researchers’ intention to test and employ their tools in areas with a higher burden of medical risk for errors [49]. The mechanism for the learning was often unclear. Teachers would find it hard to replicate the studies, as reports of costs, time commitment, clinical preparation time and resource implications were omitted.

Concerning Process factors (what happens in the teaching-for-learning moment), few papers followed a theoretical approach that could explain how the learning occurred, with some attempting to imply empirical or operational adequacy [34]. Theories are essential components for informing educational stakeholders on why and how learning takes place. Educational designs that were most helpful articulated a learning theory to show how learning takes place in practice [34, 50–52]. In the selected papers, some of the authors mentioned that their studies were informed by the educational theories, including experiential learning [27, 36–38, 41], Kolb’s Model [36], action learning [37], adult learning [38], theory of reflective practice [41]. However, they lacked the clarity on how those theories were integrated and how they work for their educational model. Likewise, the learning process did consider student preparation for observing and the use of debriefed post-observation reflection for learning. The majority of students who participated were in the mid to later years of training [27, 36–38, 42]. In one study [40], first- and second-year medical students were paired with third-year nursing students. Some of the medical students reported feeling overshadowed by the clinical experience of their inter-professional pair. The author noted that those nursing students who were already in clinical training appreciated the contextual nature of the study experience. This observation emphasised that, in the later stages of their training, students have more clinical experience to draw upon, aiding their patient safety learning from real practice. The usability of the tool was important, with some studies recommending or developing electronic methods [27, 37, 41].

The Product or outcome factors for the tools were often linked to students’ potential to monitor routine safety practices, raise safety concerns and make students feel valued by the practice team. When compared to clinical teachers, students were able to identify and categorise safety concerns but were less able to make links between patient engagement and system factors. The research evaluations mainly used a qualitative approach, and there were no experimental designs that would yield more rigorous evidence for causal associations, and thus further research is needed [53, 54]. It was encouraging to know that observational tools appeared to advance learning in all studies. Still, the evidence was not followed up by evaluating student behaviour beyond the learning event and, as such, was anecdotal. There was a limited exploration of the acceptability of the students as observers by the clinical teams, and concerns around student naivety to see the breadth of systems issues were not addressed. No study could assess the longitudinal impact of the intervention, and one study adopted a retrospective pre-post design to evaluate the learning outcome, which could have had a recall bias [38]. Despite secret observation in the Logan et al. study [39], as approved by their ethical committee, for a quality improvement project, clandestine observation could be a questionable practice in most of the hospitals.

This review has limitations. The analysis was time-limited; we could have missed papers not written in English, and we may have missed relevant studies despite following a trusted, rigorous pathway. All identified studies were based in developed, high-income countries, which restricts the generalisation and transferability to global settings, particularly less developed countries. As reviewers, we struggled with several papers that used the SBAR tool and its derivatives. Often, these papers came close to being IN but did not use the tool for real-time observational learning [55]. Those that were debated were real-time observations using SBAR in simulated settings, but not in clinical practice. While we were keen to focus on healthcare students, we justified excluding social care students, recognising that these students might have used tools of this nature and have benefited from this type of learning approach already.

## Conclusion

Observational tools used in real-time practice can benefit students’ patient safety competence, with potential benefits for raising concerns and practice standards and, thereby, benefiting patients. These types of immersive tools offer a useful mechanism for patient safety learning for pre-registration healthcare learners towards the end of their training. Learning appears to be enhanced by its experiential nature, combined with reflection, to explore the realities of real-time clinical experience. Theoretically informed studies could test out the learning mechanism, and more studies utilising theories such as Kolb, Bandura and Action theories are needed. While we found a few studies using observational tools, this learning approach requires further research to clarify setup factors and provide more theory on what works and why. In addition, more research is required to highlight how these tools, which capture real-time clinical work, can be fed back to support safe practice to benefit patient care. This paper starts a deeper discussion of how to ensure medical students learn about all aspects of patient safety when in clinical practice.

## Supplementary Information

Supplementary Material 1.

## Data Availability

No datasets were generated or analysed during the current study.
