# Supplementary material for: Exploring the use of observational tools for advancing patient safety learning among preregistration healthcare students: a scoping review using the 3P model of teaching and learning
Source: BMC Med Educ. 2026 Apr 23;26:920. doi: 10.1186/s12909-026-09275-8 (PMC13237938; doi:10.1186/s12909-026-09275-8)
Supplement: Supplementary file 1 — Supplementary Material 1. [file 12909_2026_9275_MOESM1_ESM.doc]

**Appendix I: Quality Appraisal of the Selected Papers using a Best Education Medical Education (BEME) checklist (33)**

| **Citation/ Quality domains** | **Anderson et al., 2021 (27)** | **Beekman et al., 2019 (36)** | **Bennett, 2017 (37)** | **Dundas et al., 2011 (38)** | **Logan et al., 2012 (39)** | **Spence et al., 2012 (40)** | **Steven et al., 2022 (41)** | **Thompson et al., 2016 (42)** |
| --- | --- | --- | --- | --- | --- | --- | --- | --- |
| Research Question | + | + | + | + | + | + | + | + |
| Study subject | + | + | + | + | + | + | + | + |
| Data Collection Methods | + | + | + | + | + | + | + | + |
| Completeness of Data | + | + | + | + | + | + | + | + |
| Control of Confounding | - | - | - | - | - | - | - | - |
| Analysis of Results | + | + | + | + | + | + | + | + |
| Conclusion | + | + | + | + | + | + | + | + |
| Reproducibility | + | + | + | + | + | + | + | + |
| Prospective | + | + | + | + | + | + | + | + |
| Ethical Issues | + | + | + | + | + | + | + | + |
| Triangulation | + | + | + | + | - | - | + | - |
| Positive domains | 10 | 10 | 10 | 10 | 9 | 9 | 10 | 9 |
